# Visualisation of quadratic discriminant analysis and its application in exploration of microbial interactions

**DOI:** 10.1186/s13040-015-0041-9

**Published:** 2015-02-25

**Authors:** Sugnet Gardner-Lubbe, Felix S Dube

**Affiliations:** 1 Department of Statistical Sciences, University of Cape Town, Rondebosch, 7701 Cape Town, South Africa; 2 Division of Medical Microbiology, Faculty of Health Sciences, University of Cape Town, Cape Town, South Africa

**Keywords:** Quadratic discriminant analysis, Canonical variate analysis, Biplots

## Abstract

**Background:**

When comparing diseased and non-diseased patients in order to discriminate between the aspects associated with the specific disease, it is often observed that the diseased patients have more variability than the non-diseased patients. In such cases Quadratic discriminant analysis is required which is based on the estimation of different covariance structures for the different groups. Having different covariance matrices means the Canonical variate transformation cannot be used to obtain a visual representation of the discrimination and group separation.

**Results:**

In this paper an alternative method is proposed: combining the different transformations for the different groups into a single representation of the sample points with classification regions. In order to associate the differences in variables with group discrimination, a biplot is produced which include information on the variables, samples and their relationship.

## Background

The biplot is a useful graphical method of exploring relationships in data. As the prefix ‘*bi*-’ suggests, both the samples and variables of a data matrix is represented in a biplot. The simplest form of a biplot is the Principal component analysis (PCA) biplot which optimally represents the variation in a data matrix [1]. By representing the variables on a calibrated axes [2], sample values can be read-off the axes to reveal relationships between samples and variables.

Another popular plot is a Canonical variate analysis (CVA) plot representing the optimal linear discrimination between samples from different groups, based on the assumption of equal within group variance [3]. By ensuring an aspect ratio of 1:1 is maintained and adding the original variables as calibrated biplot axes, rather than representing the canonical variates which are a mixture of the original variables, a CVA biplot is obtained. The assumption of equal within class variance allows for a single canonical transformation of all samples in all groups to a single canonical space in which the CVA biplot is constructed.

When different groups of observations have different covariance structures, the canonical transformation is not optimal for group separation. For normally distributed data, the theoretical equivalent of Linear discriminant analysis (LDA) in the presence of different group covariance matrices is Quadratic discriminant analysis (QDA).

Varying covariance structures is often found when comparing diseased to healthy patients. The variables affected by the disease have certain typical values in healthy patients. When disease sets in, the values change and change in different ways for different patients and to a different extent depending on the severity of the disease. The result is that a lot more variability is observed for the diseased patients. In an effort to understand the effect of the disease, the differences in groups are analysed by discriminant analysis. Since the covariance matrices differ, QDA can be used, but a visual representation can shed more light on the exact relationships contributing to the differences between health and disease.

In this paper a QDA biplot is suggested to visually represent the optimal separation based on respiratory pathogens in a cohort of children with suspicion of Pulmonary Tuberculosis (TB) infection. In section 2 the known and established methodology of LDA and Canonical Variate Analysis (CVA) biplots is reviewed. Section 3 deals with QDA and the QDA biplot is introduced in section 4. An example is given in section 5 before, the QDA biplot is applied to the data set of respiratory pathogens in children with TB in section 6.

## Linear discriminant analysis

We observe a set of *n* samples or observations on *p* variables, represented in the data matrix ***X***:*n × p* which we can assume without loss of generality is centred around the origin so that 1 ' ***X*** = 0 '. Of these observations, *n*_*j*_ belong to class *j*, with a total of *J* classes observed and ${\displaystyle \sum_{j=1}^J}{n}_j=n$. The class membership can be represented in a matrix ***G***:*n* × *J* with *g*_*ij*_ = 1 if sample *i* belongs to class *j* and 0 otherwise.

Fisher [4] defined LDA as a transformation that maximises the between class variance relative to the within class variance. This is closely related to CVA and multivariate analysis of variance (MANOVA) where the total variance is decomposed into a between class variance and within class variance part: ***T*** = ***B*** + ***W***, where ***T*** = ***X*** ' ***X***, ***W*** = ***X*** ' [***I*** − ***G***(***G*** ' ***G***)^− 1^***G*** ']***X***, ***B*** = ***X*** ' ***G*** ' ***GX*** and ***X*** = (***G*** ' ***G***)^− 1^***G*** ' ***X***. Fisher’s transformation to the canonical space is given by the vectors ***m***:*p* × 1 which successively maximise the ratio **(*m'Bm*) / (*m'Wm*)**. The vectors ***m*** form the columns of a matrix ***M*** which defines the transformation to canonical variates ***U*** = ***X M*** where ***M*** is the eigenvector solution to the equation ***BM***=***WMΛ*** subject to ***M'WM*** = ***I*** so that ***M*** ' ***BM*** = ***Λ*** and ***W*** = (***MM*** ')^− 1^.

The CVA biplot is constructed from the first *r*, usually *r* = 2, sometimes *r* = 3, columns of ***M***, denoted by ***M***_*r*_ and the sample points is given by ***Z*** = ***XM***_*r*_ with class means ***Z*** = ***XM***_*r*_. For more detail on the construction of the CVA biplot and fitting the biplot axes, see Gower and Hand [2] or Gower, Lubbe and le Roux [5].

Note that no assumption on the distribution of the data is made to derive the canonical transformation. However, if the data is normally distributed, such that ***X***|*G* = *j* ~ *normal*(*p*, ***μ***_*j*_, **Σ**_*W*_), the discrimination function derived at based on equal prior probability of belonging to each of the classes and equal misclassification costs for all classes is equivalent to Fisher’s LDA. The prior probabilities, i.e. the probability of belonging to class *j*, prior to observing the *p* variables in ***X***, is denoted by *π*_*j*_=*P*(*G*=*j*)s where the discrete random variable *G* should not be confused with the indicator matrix ***G***.

It is shown in Appendix A that classification of a sample is to the nearest canonical mean in the CVA biplot when the prior probabilities are equal and for unequal prior probabilities, a quantity of log(π_*j*_) is simply added to the distance to the *j*-th class mean.

## Quadratic discriminant analysis

It is assumed that the samples are random realisations from the underlying probability distributions ***X***|*G* = *j* ~ *normal*(*p*, ***μ***_*j*_, **Σ**_*j*_) where the common within class covariance matrix **Σ**_*W*_ is now replaced with *J* covariance matrices **Σ**_*j*_.

Where in LDA a sample is classified to class *k* where

$$ k=\underset{j}{\mathrm{argmax}}\left\{ \log \left({\pi}_j\right)-\frac{1}{2}\left(n-J\right)\left(\boldsymbol{u}-{\boldsymbol{u}}_j\right)\hbox{'}\left(\boldsymbol{u}-{\boldsymbol{u}}_j\right)\right\} $$

it is shown in Appendix B that classification of a sample is now to class *k* where

$$ \begin{array}{l}k= \arg \underset{j}{ \max}\left\{ \log \left({\pi}_j\right)-\frac{1}{2}\left[\left(\boldsymbol{x}-{\boldsymbol{x}}_j\right)\boldsymbol{\hbox{'}}{\boldsymbol{S}}_p^{-1}\left(\boldsymbol{x}-{\boldsymbol{x}}_j\right)+ log\left|{\boldsymbol{S}}_j\right|\right]\right\}\\ {}= \arg \underset{j}{ \max}\left\{ \log \left({\pi}_j\right)-\frac{1}{2}{\phi}_j^2\left(\boldsymbol{x}\right)\right\}\end{array} $$

Where classification for LDA was in terms of Euclidean distance in the canonical space, (***u*** − ***u***_*j*_) ' (***u*** − ***u***_*j*_), in QDA the classification function is of a similar structure, but now in terms of a function ${\phi}_j^2\left(\boldsymbol{x}\right)$.

## QDA biplot

First a simplified version is considered. Let *J* = 2 groups and the prior probabilities be equal ${\pi}_1={\pi}_2=\frac{1}{2}$. In LDA an observation ***x*** is transformed to the canonical space, ***u*** ' = ***x*** ' ***M***_*r*_ and will be classified to class 1 if (***u*** − ***u***_1_) ' (***u*** − ***u***_1_) < (***u*** − ***u***_2_) ' (***u*** − ***u***_2_) and to class 2 otherwise. The equivalent QDA classification rule will be: classify to class 1 if ${\phi}_1^2\left(\boldsymbol{x}\right)<{\phi}_2^2\left(\boldsymbol{x}\right)$. Making two different transformations $\boldsymbol{x}\to {\phi}_1^2\left(\boldsymbol{x}\right)$ and $\boldsymbol{x}\to {\phi}_2^2\left(\boldsymbol{x}\right)$ yields representations in two different one-dimensional spaces. However, plotting ${\phi}_2^2\left(\boldsymbol{x}\right)$ vs ${\phi}_1^2\left(\boldsymbol{x}\right)$ gives a two-dimensional scatter plot with the classification boundary defined by the line *y* = *x*. Since QDA is specifically applicable in cases with very different covariance structures, it will often be a feature of this plot that one group is spread out while the other is extremely concentrated, typically close to the decision boundary. A better representation can be obtained by scaling each vector ${\phi}_j^2\left(\boldsymbol{x}\right)$ to unit standard deviation. The different dimensions for plotting is already obtained by different transformations, therefore a scaling factor unique to each dimension will not add to the complexity of the representation.

Returning to the problem of *J* different classes, a different transformation is performed for each group. This creates *J* ‘new’ variables $\left({\widehat{\phi}}_j^2\left(\boldsymbol{x}\right)-{\varphi}_j\right)/{s}_j,\kern0.5em j=1, \dots,\ J$. Let these be represented in a matrix **Φ:***n* × *J*. In order to make a two-dimensional biplot, a principal component analysis on **Φ** gives the best two-dimensional representation of the *J* variables from with the transformations. The samples are represented by the first two principal components’ scores in the biplot. To construct the classification regions, each point ***z*** : 2 × 1 in the biplot space is classified to class *k* if ${\widehat{\phi}}_k^2\left(\boldsymbol{x}\right)<{\widehat{\phi}}_h^2\left(\boldsymbol{x}\right)$ for *h* = 1, …, *J*; *h* ≠ *k*. The values $$ \widehat{\phi}\hbox{'}=\left[\begin{array}{ccc}\hfill {\widehat{\phi}}_1^2\left(\boldsymbol{x}\right)\hfill & \hfill \dots \hfill & \hfill {\widehat{\phi}}_J^2\left(\boldsymbol{x}\right)\hfill \end{array}\right] $$ is obtained through back projection as described in Appendix C.

Now the plot provides a representation of the samples and classification regions. The term biplot refers to the simultaneous representation of two features of a data set, usually the samples and the variables. The plot can be enhanced to form a biplot, by adding information on the variables. Already in 1978 Kruskal and Wish [6] suggested a regression method for adding linear relationships between the samples and variables in a two dimensional display. The construction of *p*>2 variables in the display with biplot axes, rather than vectors is discussed in detail in Gower and Hand [2], Greenacre [7] and Gower, Lubbe and le Roux [5].

## An example

To illustrate the QDA biplot a simulated data set will be used. In section 3 it was mentioned that QDA is derived for data from *J* different normal distributions. Here we will use *J=*3 groups with different means and covariance matrices and 50 samples in each group.

$$ \boldsymbol{\mu} {\boldsymbol{\hbox{'}}}_1=\left[\begin{array}{cccc}\hfill 1\hfill & \hfill 1\hfill & \hfill 1\hfill & \hfill 1\hfill \end{array}\right];\kern0.5em \boldsymbol{\mu} {\boldsymbol{\hbox{'}}}_2=\left[\begin{array}{cccc}\hfill -1\hfill & \hfill 2\hfill & \hfill 3\hfill & \hfill 4\hfill \end{array}\right];\kern0.5em \boldsymbol{\mu} {\boldsymbol{\hbox{'}}}_3=\left[\begin{array}{cccc}\hfill 1\hfill & \hfill 1\hfill & \hfill 5\hfill & \hfill 5\hfill \end{array}\right] $$

$$ {\boldsymbol{\Sigma}}_1=\left[\begin{array}{cccc}\hfill 1\hfill & \hfill 0\hfill & \hfill 0\hfill & \hfill 0\hfill \\ {}\hfill 0\hfill & \hfill 1\hfill & \hfill 0\hfill & \hfill 0\hfill \\ {}\hfill 0\hfill & \hfill 0\hfill & \hfill 1\hfill & \hfill 0\hfill \\ {}\hfill 0\hfill & \hfill 0\hfill & \hfill 0\hfill & \hfill 1\hfill \end{array}\right];\kern0.5em {\boldsymbol{\Sigma}}_2=\left[\begin{array}{cccc}\hfill 2\hfill & \hfill 0\hfill & \hfill 0\hfill & \hfill 0\hfill \\ {}\hfill 0\hfill & \hfill 2\hfill & \hfill 0\hfill & \hfill 0\hfill \\ {}\hfill 0\hfill & \hfill 0\hfill & \hfill 2\hfill & \hfill 0\hfill \\ {}\hfill 0\hfill & \hfill 0\hfill & \hfill 0\hfill & \hfill 2\hfill \end{array}\right];\ {\boldsymbol{\Sigma}}_3=\left[\begin{array}{cccc}\hfill 1\hfill & \hfill 0.7\hfill & \hfill 0.7\hfill & \hfill 0.7\hfill \\ {}\hfill 0.7\hfill & \hfill 1\hfill & \hfill 0.7\hfill & \hfill 0.7\hfill \\ {}\hfill 0.7\hfill & \hfill 0.7\hfill & \hfill 1\hfill & \hfill 0.7\hfill \\ {}\hfill 0.7\hfill & \hfill 0.7\hfill & \hfill 0.7\hfill & \hfill 1\hfill \end{array}\right] $$

**Figure 1:**
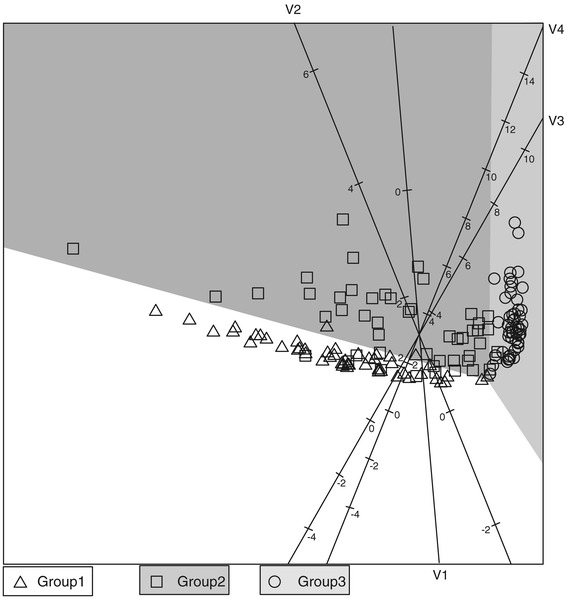
**QDA biplot of simulated data from a normal distribution.**

The QDA biplot is given in Figure 1. Since simulated data was used, the features of the data are known and it is clear that these features are well represented in the QDA biplot. We have $$ {\boldsymbol{\mu}}_1^{\hbox{'}}=\left[\begin{array}{cccc}\hfill 1\hfill & \hfill 1\hfill & \hfill 1\hfill & \hfill 1\hfill \end{array}\right] $$ which has the lowest values for variables 2, 3 and 4 than Groups 2 and 3. From the biplot we see that group 1 has lower values for all variables except variable 1. Group 2 has more variation that the other two groups which is consistent with the diagonal values of **Σ**_2_, and lies between Groups 1 and 3. Orthogonally projecting onto the axes of variables 3 and 4, it is clear that Group 3 has the highest values, consistent with *μ*_33_ = *μ*_34_ = 5.

In the example above, the data was simulated from a normal distribution so it is known that the QDA methodology is applicable to the specific data set. However, the application of respiratory pathogens contains only indicator variables with 0 = absence and 1 = presence of the pathogen. Before applying the QDA biplot on this data set, the simulated data set is converted into indicator variables with all values less than the median zero and all values larger than or equal to the median being made one. Categorising the data will lead to a loss of information, but we expect some degree of similarity in location and spread between the normally distributed data set and the indicator variable data set. The degree to which the QDA biplots of the two data sets represent the same location, spread and separation features will give an indication of how well the QDA biplot performs in cases where the data does not follow a normal distribution.

**Figure 2:**
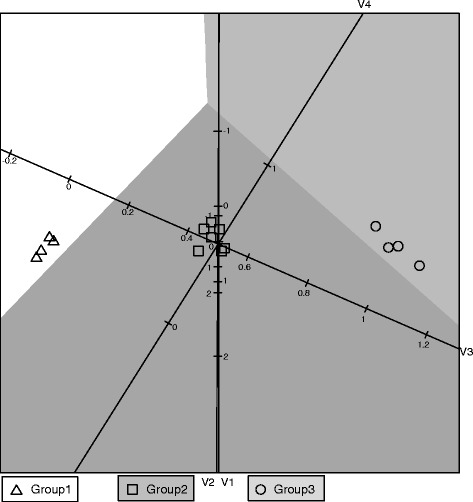
**QDA biplot of simulated indicator data.**

The QDA biplot of the indicator variable data set is given in Figure 2. With four variables which can each only take on one of two values (0 or 1), there is only 2^4^ = 16 different response patterns possible. In the simulated data set, 15 of the 16 patterns occurred at least once. All identical patterns will be on the same point in the biplot. For each point the symbol displayed is found by majority vote. The same problem with different response patterns does not occur in the application in section 6 since a total of 15 pathogens yields 16,384 different response patterns.

In the QDA biplot in Figure 2 it is clear that the majority of the samples appear in their correct classification regions. This was also the case in Figure 1. The small differences in variable 2 disappear with the course coding and the three groups appear to be similar on variables 1 and 2. Group 1 has the lowest values (most zero’s) for variables 3 and 4 while Group 3 has the highest values (most one’s) for variables 3 and 4. Again Group 2 appears to be located between Groups 1 and 3. It is comforting to see that the primary location, spread and separation features of the data set did not change between Figures 1 and 2, although converting the data to indicator variables did lead to a loss of information. Moore [8] evaluates discrimination procedures for binary data. Here the focus is on obtaining a visualisation of how the variables relate to the different groups when separating groups with differences in covariance structure. The comparison of Figures 1 and 2 shows that the biplot remains a useful tool for exploring the variables contributing to differences between groups with unequal covariance matrices.

## Application: Distribution of respiratory pathogens in a cohort of children with suspicion of pulmonary tuberculosis infection

**Figure 3:**
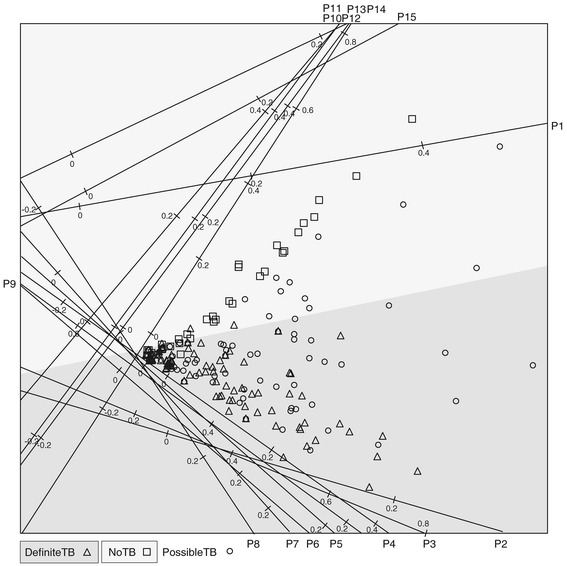
**QDA biplot of the pathogen data with linear biplot axes and classification regions based on prior probabilities proportional to the sample size.**

In this section the QDA biplot will be illustrated with the data set that inspired the development of the plot. Medical researchers were interested in examining the distribution of respiratory pathogens detected in respiratory specimens from children presenting for care with symptoms suggestive of pulmonary tuberculosis. The children are classified into one of three groups: definite-TB (microbiologically confirmed), non-TB (microbiologically confirmed) and possible-TB (microbiologically excluded). Detailed microbiological methods are published elsewhere (In Press). Among other analyses, QDA was performed on the definite and no-TB groups since the possible-TB patients are actually unclassified members of the former two groups. The principal interest of the researchers is to associate some pathogens with the clinical manifestation of definite-TB and some with no-TB. The QDA biplot is given in Figure 3. The method of orthogonal parallel translation of the biplot axes as detailed in Gower, Lubbe and le Roux [5] was applied to move the biplot axes out of the way of the samples to obtain a clearer plot.

Since the primary interest of this analysis is not classification, the biplot is a useful tool to visualize how the variables relate to the definite and no-TB groups. Pathogens 2 to 9 are all associated with TB while pathogens 10, 11, 13 and 14 are associated with the no-TB group. Pathogens 1, 12 and 15 seem to have a mixture of definite and no-TB patients. The spread of the sample points from zero on the left towards higher pathogen values in a triangle shape show that for the definite-TB group some patients have little, if any, of the pathogens while some others have some combination of pathogens 2 to 8. Pathogen 9 is the exception which seems to be negatively correlated with pathogens 2 to 8. Similarly, some no-TB patients have few or no of pathogens 10, 11, 13 or 14 while others have a combination of these.

In a pilot study, the visual aid of the biplot provides an easily understandable aid to which pathogens relate to which of the two groups. Actually a total of 33 pathogens were measured, but those not really contributing to the discrimination between definite-TB and no-TB are not shown here.

## Conclusion

In cases where the variance between groups differs, QDA should be applied with the estimation of different covariance matrices for different groups. A transformation based on the optimal classification of samples from normal distributions is suggested to construct a QDA biplot. In the biplot both the samples, with classification regions, and original variables are represented, showing the relationships between different groups and the various variables.

Through a simple simulation, it was verified that the main characteristics of the plot remains intact, even if the assumption of normality is not justified.

The QDA biplot is not designed in the first place for optimal classification of samples, as this can be performed algebraically with many software programmes. The main purpose of the QDA biplot is to provide a visual representation of the relationships between samples in a specific group and the variables measured.

## Appendix A: Linear Discriminant Analysis

For classification of an object the posterior probability of belonging to each of the *J* groups is calculated, *π*_*j*|***x***_ = *π*_*j*_*f*_***X***|*G*_(***x***|*G* = *j*), and the sample is classified to the group with largest posterior probability, $\arg \underset{j}{ \max }{\pi}_{j\Big|\boldsymbol{x}}$. The posterior probabilities needs to be estimated from the observed data and for the methodology applied in sections 3 and 4, it is important to look at the log odds of the estimated posterior probabilities. Using the estimates ***x***_*j*_ and pooled sample covariance matrix ***S***_*p*_ a sample is classified to

$$ class\ J\  if\  log\left(\frac{{\widehat{\pi}}_{j\Big|\boldsymbol{x}}}{{\widehat{\pi}}_{J\Big|\boldsymbol{x}}}\right)<0,\kern0.5em j=1, \dots,\ J-1 $$

$$ class\ k\  if\  log\left(\frac{{\widehat{\pi}}_{j\Big|\boldsymbol{x}}}{{\widehat{\pi}}_{J\Big|\boldsymbol{x}}}\right)< log\left(\frac{{\widehat{\pi}}_{k\Big|\boldsymbol{x}}}{{\widehat{\pi}}_{J\Big|\boldsymbol{x}}}\right),\kern0.5em j=1, \dots,\ J-1;j\ne k $$

where the log odds can be written as

$$ \begin{array}{l}g\left(\frac{{\widehat{\pi}}_{j\Big|\boldsymbol{x}}}{{\widehat{\pi}}_{J\Big|\boldsymbol{x}}}\right)= log\left(\frac{\pi_j}{\pi_J}\right)+ log\left(\frac{{\left(2\pi \right)}^{-\frac{p}{2}}{\left|{\boldsymbol{S}}_p\right|}^{-\frac{1}{2}} exp\left[-\frac{1}{2}\left(\boldsymbol{x}-{\boldsymbol{x}}_j\right)\hbox{'}{\boldsymbol{S}}_p^{-1}\left(\boldsymbol{x}-{\boldsymbol{x}}_j\right)\right]}{{\left(2\pi \right)}^{-\frac{p}{2}}{\left|{\boldsymbol{S}}_p\right|}^{-\frac{1}{2}} exp\left[-\frac{1}{2}\left(\boldsymbol{x}-{\boldsymbol{x}}_J\right)\hbox{'}{\boldsymbol{S}}_p^{-1}\left(\boldsymbol{x}-{\boldsymbol{x}}_J\right)\right]}\right)\\ {}= log\left(\frac{\pi_j}{\pi_J}\right)-\frac{1}{2}\left\{{\delta}^2\left(\boldsymbol{x},\ {\boldsymbol{x}}_j,\ {\boldsymbol{S}}_p\right)-{\delta}^2\left(\boldsymbol{x},\ {\boldsymbol{x}}_J,\ {\boldsymbol{S}}_p\right)\right\}\end{array} $$

with $$ {\delta}^2\left(\boldsymbol{x},\ {\boldsymbol{x}}_j,\ {\boldsymbol{S}}_p\right)={\left(\boldsymbol{x}-{\boldsymbol{x}}_j\right)}^{\hbox{'}}{\boldsymbol{S}}_p^{-1}\left(\boldsymbol{x}-{\boldsymbol{x}}_j\right) $$.

This means that a sample is classified to group *k* where

$$ {\boldsymbol{S}}_p=\frac{{\displaystyle {\sum}_j}\left({n}_j-1\right){\boldsymbol{S}}_j}{{\displaystyle {\sum}_j}\left({n}_j-1\right)}=\frac{{\boldsymbol{X}}^{\boldsymbol{\hbox{'}}}\boldsymbol{X}-\boldsymbol{X}\boldsymbol{\hbox{'}}\boldsymbol{G}{\left({\boldsymbol{G}}^{\boldsymbol{\hbox{'}}}\boldsymbol{G}\right)}^{-1}\boldsymbol{G}\boldsymbol{\hbox{'}}\boldsymbol{X}}{{\displaystyle {\sum}_j}\left({n}_j-1\right)}==\frac{1}{{\displaystyle {\sum}_j}\left({n}_j-1\right)}\boldsymbol{W} $$

and

$$ \begin{array}{l}k=\underset{j}{\mathrm{argmax}}\left\{ \log \left({\pi}_j\right)-\frac{1}{2}{\delta}^2\left(\boldsymbol{x},\ {\boldsymbol{x}}_j,\ {\boldsymbol{S}}_p\right)\right\}=\underset{j}{\mathrm{argmax}}\left\{ \log \left({\pi}_j\right)-\frac{1}{2}\left(n-J\right)\left(\boldsymbol{x}-{\boldsymbol{x}}_j\right)\hbox{'}{\boldsymbol{W}}^{-1}\left(\boldsymbol{x}-{\boldsymbol{x}}_j\right)\right\}\\ {}=\underset{j}{\mathrm{argmax}}\left\{ \log \left({\pi}_j\right)-\frac{1}{2}\left(n-J\right)\left(\boldsymbol{x}-{\boldsymbol{x}}_j\right)\hbox{'}\boldsymbol{M}\boldsymbol{M}\hbox{'}\left(\boldsymbol{x}-{\boldsymbol{x}}_j\right)\right\}\\ {}=\underset{j}{\mathrm{argmax}}\left\{ \log \left({\pi}_j\right)-\frac{1}{2}\left(n-J\right)\left(\boldsymbol{u}-{\boldsymbol{u}}_j\right)\hbox{'}\left(\boldsymbol{u}-{\boldsymbol{u}}_j\right)\right\}\end{array} $$

so that classification is to the nearest canonical mean in the CVA biplot, barring an additive factor depending on the prior probability. Should the prior probabilities all be equal, classification is simply to the nearest class mean in the CVA biplot.

## Appendix B

For classification a sample is classified to the group with largest posterior probability where a sample is classified to

$$ class\ J\  if\  log\left(\frac{{\widehat{\pi}}_{j\Big|\boldsymbol{x}}}{{\widehat{\pi}}_{J\Big|\boldsymbol{x}}}\right)<0,\kern0.5em j=1, \dots,\ J-1 $$

$$ class\ k\  if\  log\left(\frac{{\widehat{\pi}}_{j\Big|\boldsymbol{x}}}{{\widehat{\pi}}_{J\Big|\boldsymbol{x}}}\right)< log\left(\frac{{\widehat{\pi}}_{k\Big|\boldsymbol{x}}}{{\widehat{\pi}}_{J\Big|\boldsymbol{x}}}\right),\kern0.5em j=1, \dots,\ J-1;j\ne k $$

where the log odds can be written as

$$ \begin{array}{l} log\left(\frac{{\widehat{\pi}}_{j\Big|\boldsymbol{x}}}{{\widehat{\pi}}_{J\Big|\boldsymbol{x}}}\right)= log\left(\frac{\pi_j}{\pi_J}\right)+ log\left(\frac{{\left(2\pi \right)}^{-\frac{p}{2}}{\left|{\boldsymbol{S}}_j\right|}^{-\frac{1}{2}} exp\left[-\frac{1}{2}\left(\boldsymbol{x}-{\boldsymbol{x}}_j\right)\hbox{'}{\boldsymbol{S}}_j^{-1}\left(\boldsymbol{x}-{\boldsymbol{x}}_j\right)\right]}{{\left(2\pi \right)}^{-\frac{p}{2}}{\left|{\boldsymbol{S}}_J\right|}^{-\frac{1}{2}} exp\left[-\frac{1}{2}\left(\boldsymbol{x}-{\boldsymbol{x}}_J\right){\boldsymbol{S}}_J^{-1}\left(\boldsymbol{x}-{\boldsymbol{x}}_J\right)\right]}\right)\\ {}= log\left(\frac{\pi_j}{\pi_J}\right)-\frac{1}{2}\left\{ log\left(\frac{\left|{\boldsymbol{S}}_j\right|}{\left|{\boldsymbol{S}}_J\right|}\right)+{\delta}^2\left(\boldsymbol{x},\ {\boldsymbol{x}}_j,\ {\boldsymbol{S}}_j\right)-{\delta}^2\left(\boldsymbol{x},\ {\boldsymbol{x}}_J,\ {\boldsymbol{S}}_J\right)\right\}\end{array} $$

with $$ {\delta}^2\left(\boldsymbol{x},\ {\boldsymbol{x}}_j,\ {\boldsymbol{S}}_j\right)=\left(\boldsymbol{x}-{\boldsymbol{x}}_j\right)\hbox{'}{\boldsymbol{S}}_j^{-1}\left(\boldsymbol{x}-{\boldsymbol{x}}_j\right) $$.

Define $$ {\phi}_j^2\left(\boldsymbol{x}\right)={\delta}^2\left(\boldsymbol{x},\ {\boldsymbol{x}}_j,\ {\boldsymbol{S}}_j\right)+ log\left|{\boldsymbol{S}}_j\right| $$, then

$$ log\left(\frac{\pi_{j\Big|\boldsymbol{x}}}{\pi_{J\Big|\boldsymbol{x}}}\right)= log\left(\frac{\pi_j}{\pi_J}\right)-\frac{1}{2}\left\{{\phi}_j^2\left(\boldsymbol{x}\right)-{\phi}_J^2\left(\boldsymbol{x}\right)\right\} $$

and the sample ***x*** is classified to the group with largest posterior probability, $\arg \underset{j}{ \max}\left\{ \log \left({\pi}_j\right)-\frac{1}{2}{\phi}_j^2\left(\boldsymbol{x}\right)\right\}$.

## Appendix C: Back projection in PCA

Although PCA is always performed on a centred data matrix, it was argued in section 4 that the values in the matrix $$ \left[\begin{array}{ccc}\hfill {\phi}_1^2\left({\boldsymbol{x}}_{(1)}\right)\hfill & \hfill \dots \hfill & \hfill {\phi}_J^2\left({\boldsymbol{x}}_{(1)}\right)\hfill \\ {}\hfill \vdots \hfill & \hfill \ddots \hfill & \hfill \vdots \hfill \\ {}\hfill {\phi}_1^2\left({\boldsymbol{x}}_{(n)}\right)\hfill & \hfill \dots \hfill & \hfill {\phi}_J^2\left({\boldsymbol{x}}_{(n)}\right)\hfill \end{array}\right]:n\times J $$ should also be standardised by dividing each column by its standard deviation. Let ***φ*** : *J* × 1 and ***s*** : *J* × 1 represent the column means and sample standard deviations then PCA is performed on the matrix

$$ \boldsymbol{\Phi} =\left(\left[\begin{array}{ccc}\hfill {\phi}_1^2\left({\boldsymbol{x}}_{(1)}\right)\hfill & \hfill \dots \hfill & \hfill {\phi}_J^2\left({\boldsymbol{x}}_{(1)}\right)\hfill \\ {}\hfill \vdots \hfill & \hfill \ddots \hfill & \hfill \vdots \hfill \\ {}\hfill {\phi}_1^2\left({\boldsymbol{x}}_{(n)}\right)\hfill & \hfill \dots \hfill & \hfill {\phi}_J^2\left({\boldsymbol{x}}_{(n)}\right)\hfill \end{array}\right]-1\left[\begin{array}{ccc}\hfill {\varphi}_1\hfill & \hfill \dots \hfill & \hfill {\varphi}_J\hfill \end{array}\right]\right)\left[\begin{array}{cccc}\hfill {s}_1^{-1}\hfill & \hfill 0\hfill & \hfill \dots \hfill & \hfill 0\hfill \\ {}\hfill \vdots \hfill & \hfill \ddots \hfill & \hfill \dots \hfill & \hfill 0\hfill \\ {}\hfill 0\hfill & \hfill 0\hfill & \hfill \dots \hfill & \hfill {s}_J^{-1}\hfill \end{array}\right] $$

with singular value decomposition

$$ \boldsymbol{\Phi} =\boldsymbol{U}\boldsymbol{D}\boldsymbol{V}\hbox{'} $$

The principal component scores for the first two dimensions is obtained from the first two columns of the matrix ***V***, $$ \left[\begin{array}{cc}\hfill {\boldsymbol{v}}_1\hfill & \hfill {\boldsymbol{v}}_2\hfill \end{array}\right]={\boldsymbol{V}}_2:J\times 2 $$:

$$ \boldsymbol{Z}:n\times 2=\boldsymbol{\Phi} {\mathbf{V}}_2 $$

and the back projections is given by

$$ \widehat{\varPhi}=\boldsymbol{Z}\boldsymbol{V}{\hbox{'}}_2=\boldsymbol{\Phi} {\boldsymbol{V}}_2\boldsymbol{V}{\hbox{'}}_2 $$

as shown in Gower and Hand [2]. To obtain the back projected value for the unscaled, uncentred ${\phi}_j^2\left({\boldsymbol{x}}_{(i)}\right)$ -value, the operations are reversed to give

$$ {\widehat{\phi}}_j^2\left({\boldsymbol{x}}_{(i)}\right)={\widehat{\phi}}_{ij}{s}_j+{\varphi}_j $$

where ${\widehat{\phi}}_{ij}$ is the *ij* -th element of the matrix $\widehat{\varPhi}$. ***z*****’*****V***_2_^’^*diag*(*s*_1_, …, *s*_*J*_) + ***φ***
